# A Hybrid Monkey Search Algorithm for Clustering Analysis

**DOI:** 10.1155/2014/938239

**Published:** 2014-03-04

**Authors:** Xin Chen, Yongquan Zhou, Qifang Luo

**Affiliations:** ^1^College of Information Science and Engineering, Guangxi University for Nationalities, Nanning Guangxi 530006, China; ^2^Guangxi Key Laboratory of Hybrid Computation and Integrated Circuit Design Analysis, Nanning Guangxi 530006, China

## Abstract

Clustering is a popular data analysis and data mining technique. The *k*-means clustering algorithm is one of the most commonly used methods. However, it highly depends on the initial solution and is easy to fall into local optimum solution. In view of the disadvantages of the *k*-means method, this paper proposed a hybrid monkey algorithm based on search operator of artificial bee colony algorithm for clustering analysis and experiment on synthetic and real life datasets to show that the algorithm has a good performance than that of the basic monkey algorithm for clustering analysis.

## 1. Introduction

Cluster analysis or clustering is the task of grouping a set of objects in such a way that objects in the same group (called cluster) are more similar (in some sense or another) to each other than to those in other groups (clusters). It is a main task of exploratory data mining and a common technique for statistical data analysis used in many fields, including machine learning, pattern recognition, image analysis, information retrieval, and bioinformatics. Cluster analysis was originated in anthropology by Driver and Kroeber in 1932 and introduced to psychology by Zubin in 1938 and Tryon in 1939 and famously used by Cattell beginning of 1943 [1] for trait theory classification in personality psychology. Many clustering methods have been proposed; it is divided into two main categories: hierarchical and partitional. The *k*-means clustering method [2] is one of the most commonly used partitional methods. However the results of *k*-means solving the clustering problem highly depend on the initial solution and it is easy to fall into local optimal solutions. Zhang et al. have proposed an improved *k*-means clustering algorithm called *k*-harmonic means [3]. But the accuracy of the results obtained by the method is not high.

In order to overcome this problem, many scholars began to solve the problem using metaheuristic algorithms. In 1991, Colorni et al. have presented ant colony optimization (ACO) algorithm based on the behavior of ants seeking a path between their colony and a source of food. Then Shelokar et al. and Kao and Cheng solved the clustering problem using the ACO algorithm [4, 5]. Niknam et al. have proposed an efficient hybrid evolutionary algorithm based on combining ACO and SA (simulated annealing algorithm, 1989 [6]) for clustering problem [7, 8]. Kennedy and Eberhart have proposed particle swarm optimizer (PSO) algorithm which simulates the movement of organisms in a bird flock or fish school in 1995 [9]. The algorithm also has been adopted to solve this problem by Omran et al. and Merwe and Engelbrecht [10, 11]. Kao et al. have presented a hybrid approach according to combination of the *k*-means algorithm, Nelder-Mead simplex search, and PSO for clustering analysis [12]. Niknam et al. have presented a hybrid evolutionary algorithm based on PSO and SA to solve the clustering problem [13]. Niknam has proposed an efficient hybrid approach based on PSO, ACO, and *k*-means called PSO-ACO-K approach for cluster analysis [14]. In 2005, the artificial bee colony (ABC) algorithm is described by Karaboga [15] and it has been adopted to solve this problem by Karaboga and Ozturk [16]. Zou et al. have proposed a cooperative artificial bee colony algorithm to solve the clustering problem and experiment on synthetic and real life datasets to evaluate the performance [17]. Voges and Pope have used an evolutionary-based rough clustering algorithm for the clustering problem [18].

Monkey algorithm (MA) is a new type of swarm intelligent algorithm. It was put forward by Ruiqing and Wansheng [19] in 2008 which is used in solving large-scale, multimodal optimization problem. The method derives from the simulation of mountain-climbing processes of monkeys. It consists of three processes: climb process, watch-jump process, and somersault process. In the original MA, the time consumed mainly lies in using the climb process to search local optimal solutions. The essential feature of this process is the calculation of the pseudogradient of the objective function that only requires two measurements of the objective function regardless of the dimension of the optimization problem. The purpose of the somersault process is to make monkeys find new search domains and this action primely avoids running into local search. Therefore, MA has been successfully applied to solve various optimization problems, such as the transmission network expansion planning [20], the intrusion detection technology [21], the optimal sensor placement in structural health monitoring [22], and the optimization of gas filling station project scheduling problem [23]. In view of the characteristics of the clustering problem, this paper proposed a monkey algorithm with search operator of artificial bee colony algorithm (ABC-MA). The algorithm introduced the ABC search operator before the climb process to strengthen the local search ability and to improve the somersault process combined with the *k*-means method. The algorithm improves the calculation accuracy in a certain degree. The numerical experiment results show that the proposed algorithm has good performance than that of the basic monkey algorithm for solving the clustering problem.

## 2. The *k*-Means Clustering Algorithm

The goal of data clustering is grouping data into a number of clusters. *k*-means is one of the simplest unsupervised learning algorithms that solve the well-known clustering problem. It was proposed by MacQueen in 1967 [24]. The procedure follows a simple and easy way to classify a given data set *D* = {*x*_1_, *x*_2_,…, *x*_*n*_} through a certain number of clusters *G*_1_, *G*_2_ …, *G*_*K*_ (assume *K* clusters) fixed a priori; each data vector is a *p*-dimensional vector, satisfying the following conditions [25, 26]:

1. *G*_*i*_ ≠ *∅*, *i* = 1,2,…*K*;
2. *G*_*i*_∩*G*_*j*_ = *∅*, *i*, *j* = 1,2,…, *K*, *i* ≠ *j*;
3. ⋃_*i*=1_^*K*^ *G*_*i*_ = {*x*_1_, *x*_2_,…, *x*_*n*_}.

The *k*-means clustering algorithm is as follows.

(1) Set the number of clusters *K* and the data set *D* = {*x*_1_, *x*_2_,…, *x*_*n*_}.
(2) Randomly choose *K* points *c*_1_, *c*_2_,…, *c*_*K*_ as the cluster centroids from {*x*_1_, *x*_2_,…, *x*_*n*_}.
(3) Assign each object *x*_*j*_ to the group that has the closest centroid. The principle of division is as follows: if *d*(*x*_*i*_ − *c*_*j*_) < *d*(*x*_*i*_ − *c*_*k*_), *k* = 1,2,…, *K* and *j* ≠ *k*. The data *x*_*i*_ will be divided into classified collection *G*_*j*_.
(4) When all objects have been assigned, recalculate the positions of the *K* centroids *c*_1_*, *c*_2_*,…, *c*_K_*:
  (1) ci∗=1|Gi|∑xj∈Gjxj, i=1,2,…K,
 where |*G*_*i*_| is the number of the points in the classified collection *G*_*j*_.
(5) Repeat steps 2 and 4 until the centroids no longer move.

The main idea is to define *K* centroids, one for each cluster. These centroids should be placed in a cunning way because a different location causes different result. So, the better choice is to place them as much as possible far away from each other. In this study, we will use Euclidian metric as a distance metric. The expression is given as follows:

(2) d(xi,cj)=∑k=1p(xik−cjk)2.

Finally, this algorithm aims at minimizing an objective function, in this case, a squared error function. The objective function

(3) f(X,C)=∑i=1nmin⁡{||xi−ck||2 ∣ k=1,2,…,p}.

## 3. Description of Modified Monkey Algorithm

The MA is a novel kind of evolutionary algorithm which can solve a variety of difficult optimization problems featuring nonlinearity, nondifferentiability, and high dimensionality. The difference from the other algorithms is that the time consumed by the MA mainly lies in using the climb process to search local optimal solutions. So according to the characteristics of the clustering problem, a new monkey algorithm with the search operator of artificial bee colony is proposed. In this section, we mainly describe the main components of the algorithm, representation of solution, initialization, climb process, watch-jump process, and improved somersault process and search operator. The details are listed as follows.

### 3.1. Representation of Solution

At first an integer *M* is defined as the population size of monkeys. And then, for the monkey *i*, its position is denoted as a vector *X*_*i*_ = (*x*_*i*,1_, *x*_*i*,2_,…, *x*_*i*,*K*∗*p*_), where *K* is equal to the number of the cluster centroids, and each cluster centroid includes *p* components. The position will be employed to express a solution of the optimization problem.

### 3.2. Initial Population

Initialization of the population will have great effect on the precision. In the original MA, the initial populations of possible solutions are generated randomly in the solution interval. However, for the clustering problem, each component of the data has different intervals. So, for monkey *i*, we randomly choose *K* of the samples (each sample includes *p* components) from the data set.

### 3.3. Climb Process

The climb process is a step-by-step procedure to change the monkeys' positions from the initial positions to new ones that can make an improvement in the objective function. The climb process is designed to use the idea of pseudo-gradient-based simultaneous perturbation stochastic approximation (SPSA) [27, 28], a kind of recursive optimization algorithm. For the monkey *i*, its position is *X*_*i*_ = (*x*_*i*,1_, *x*_*i*,2_,…, *x*_*i*,*K*∗*p*_), *i* = 1,2,…, *M*, respectively. *f*(*X*_*i*_) is the corresponding fitness value. The improved climb process is given as follows.

 (1) Randomly generate two vectors Δ*x*_*i*_ = (Δ*x*_*i*,1_, Δ*x*_*i*,2_,…, Δ*x*_*i*,*K*∗*p*_), where
  (4) Δxij={awith  probability  12−awith  probability  12
 
*j* = 1,2,…, *K*∗*p*, respectively. The parameter *a* (*a* > 0), called the step of the climb process, can be determined by specific situations. The step length *a* plays a crucial role in the precision of the approximation of the local solution in the climb process. Usually, the smaller the parameter *a* is, the more precise the solutions are.
 (2) Calculate
  (5) fij′(Xi)=f(Xi+Δxi)−f(Xi−Δxi)2Δxij,
 
*j* = 1,2,…, *K*∗*p*, respectively. The vector *f*_*ij*_′(*X*_*i*_) = (*f*_*i*,1_′(*X*_*i*_), *f*_*i*,2_′(*X*_*i*_),…, *f*_*i*,*K*∗*p*_′(*X*_*i*_)) is called the pseudogradient of the objective function at the point *X*_*i*_.
 (3) Set *y*_*j*_ = *x*_*ij*_ + *a* · sign⁡(*f*_*ij*_′(*X*_*i*_)), *j* = 1,2,…, *K*∗*p*, respectively, and let *Y* = (*y*_1_, *y*_2_,…, *y*_*K*∗*p*_).
 (4) Update *X*_*i*_ with *Y* provided that *Y* is feasible. Otherwise, we keep *X*_*i*_ unchanged.
 (5) Repeat steps (1) to (4) until the maximum allowable number of iterations (called the climb number, denoted by *Nc*) has been reached.

**Figure 1:**
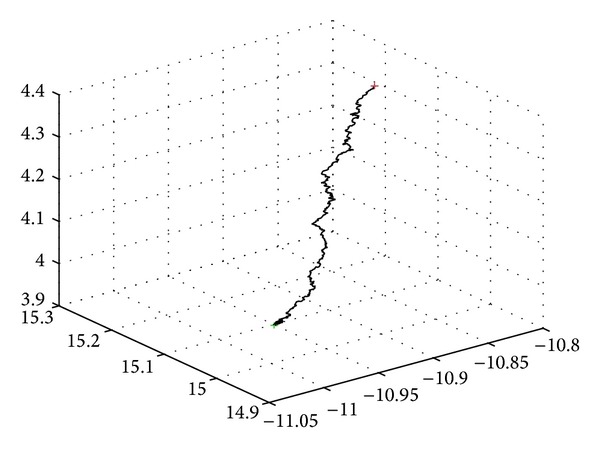
The climb process with climb step 0.001 and climb number 1000 for solving *f*(*x*) = *x* ^2^ in the 3d space.

Figure 1 shows the climb process of the monkey seeking the local optimal solution of *f*(*x*) = *x* ^2^ with climb step 0.001 and climb number 1000 in 3d space. The red point represents the initial position and the green is the end.

### 3.4. Watch-Jump Process

After the climb process, each monkey arrives at its own mountaintop. And then it will take a look and determine whether there are other points around it being higher than the current one. If yes, it will jump there from the current position and then repeat the climb process until it reaches the top of the mountain. For the monkey *i*, its position is *X*_*i*_ = (*x*_*i*,1_, *x*_*i*,2_,…, *x*_*i*,*K*∗*p*_), *i* = 1,2,…, *M*. The watch-jump process is given as follows.

1. Randomly generate real numbers *y*_*j*_ from (*x*_*ij*_ − *b*, *x*_*ij*_ + *b*), *j* = 1,2,…, *K*∗*p*, respectively. Let *Y* = (*y*_1_, *y*_2_,…, *y*_*K*∗*p*_). The parameter *b* is called the eyesight of monkeys which can be determined by specific situations. Usually, the bigger the feasible space of optimal problem is, the bigger the value of *b* should be taken.
2. Update *X*_*i*_ with *Y* provided that both *f*(*Y*) ≥ *f*(*X*_*i*_) and *Y* are feasible. Otherwise, repeat step (1) until an appropriate point *Y* is found. For the clustering problem, we only replace *X*_*i*_ with *Y* whose function value is smaller than or equal to *f*(*X*_*i*_).
3. Repeat the climb process by employing *Y* as an initial position.

### 3.5. Somersault Process Based on the *k*-Means

**Figure 2:**
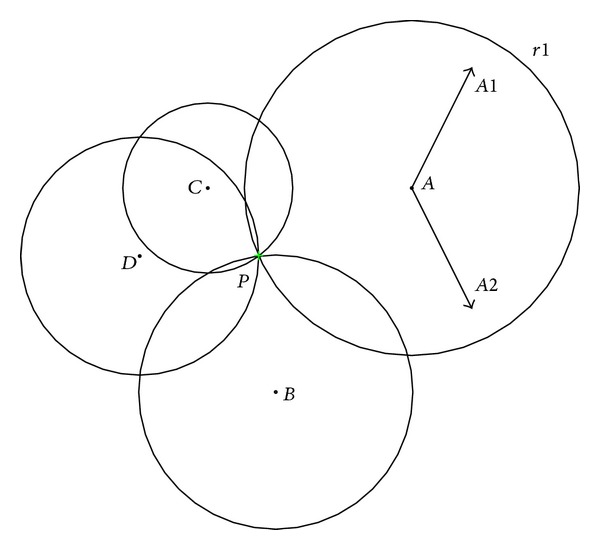
The somersault process of the original MA.

After repetitions of the climb process and the watch-jump process, each monkey will find a locally maximal mountaintop around its initial point. In order to find a much higher mountaintop, it is natural for each monkey to somersault to a new search domain. In the original MA, the monkeys will somersault along the direction pointing to the pivot which is equal to the bar center of all monkeys' current positions.  Figure 2 shows the somersault process of the original MA [19]. The points *A*, *B*, *C*, and *D* represent monkeys. The point *P* is the center of all monkeys, the somersault interval [*c*, *d*] = [−1,1]. For example, the monkey *A* can reach any point (such as points *P*, *A*1, and *A*2) within the circle *r*1 because of the somersault interval [*c*, *d*] = [−1,1].

However, the monkey is easy to leave the solution interval for the clustering problem and all monkeys will lose the population diversity because of somersaulting along the direction pointing the pivot after many iterations. Here we choose the center of objects belonging to the cluster as the pivot to replace the center of all monkeys by the *k*-means algorithm. For the monkey *i*, its position is *X*_*i*_ = (*x*_*i*,1_, *x*_*i*,2_,…, *x*_*i*,*K*∗*p*_); the improved somersault process is given as follows.

(1) Assign each object to the group that has the closest centroid *G*_1_, *G*_2_,…, *G*_*K*_ according to the location of the monkey *i*.
(2) Randomly generate real numbers *θ* from the interval [*c*, *d*] (called the somersault interval, which decides the maximum distance that monkeys can somersault).
(3) Calculation the *K* positions *c*_1_*, *c*_2_*,…, *c*_K_* which are the centers of objectives belonging to centroid *G*_1_, *G*_2_,…, *G*_*K*_ according to the formula (1), respectively. The *K* positions form a vector which represents the pivot to replace the center of monkeys. Let *c* = (*c*_1_*, *c*_2_*,…, *c*_K_*) = (*c*_1_, *c*_2_,…, *c*_*K*∗*p*_).
(4) Set
  (6) yj=xij+θ(cj−xij),
 
*j* = 1,2,…, *K*∗*p*, respectively.
(5) Update *X*_*i*_ with *Y* provided that both *f*(*Y*) ≥ *f*(*X*_*i*_) and *Y* are feasible. Otherwise, generate a new solution to replace *X*_*i*_.

### 3.6. Search Operator

The original MA mainly lies in using the climb process to search local optimal solutions. The climb step plays a crucial role in the precision of the approximation of the local solution. The smaller the climb step is, the bigger the climb number is and the higher precision the solution is; it will spend a lot of time to calculate the objective value. For example, the climb step is 0.01; the climb number should be set 100, so it needs to calculate 200 times objective function value every climb process. When we set the climb step 0.001, the climb number should be set 1000; we need to calculate 2000 times objective function value every climb process. In order to reduce the computing time, this paper introduced search operator of artificial bee colony algorithm before climb process.

The artificial bee colony optimization algorithm (ABC) is described by Karaboga based on the foraging behavior of honey bees [29]. In the ABC, the colony consists of three groups of bees: employed bees, onlookers, and scouts. Each employed bee seeks a food source according to the search operator (7) nearby its current food source then evaluates its nectar amount and determines whether to update the food source by greedy strategy. After all employed bees complete the search process, they share the position information of the food sources with the onlookers on the dance area. Each onlooker watches the dance of employed bees and chooses one of their sources with a probability depending on the nectar amounts of sources. If a food source cannot be improved through predetermined cycles, called “limit,” it is removed from the population, and the employed bee of that food source becomes scout. The search operator of employed bees is as follow:

(7) zij=xij+ϕij(xij−xkj),

where *k* ∈ {1,2,…, *M*} and *j* ∈ {1,2,…, *p*∗*K*} are randomly chosen indexes. Although *k* is determined randomly, and it is different from *i*, *ϕ*_*ij*_ is a random number between [−1, 1]. The experimental results show that it has a good optimization performance in optimizing complex multimodal problems [29] due to the strong local exploration ability of search operator.

In the MA, the local exploration ability of the climb process is weak and the somersault process has strong global search ability. Here we introduced the ABC search operator before the climb process to strengthen seeking the local optimal solution. For each monkey, each component is updated once adopting the ABC search operator. So each monkey will move *p*∗*K* times. The local search process before the climb process is as shown in Algorithm 1.

**Figure 3:**
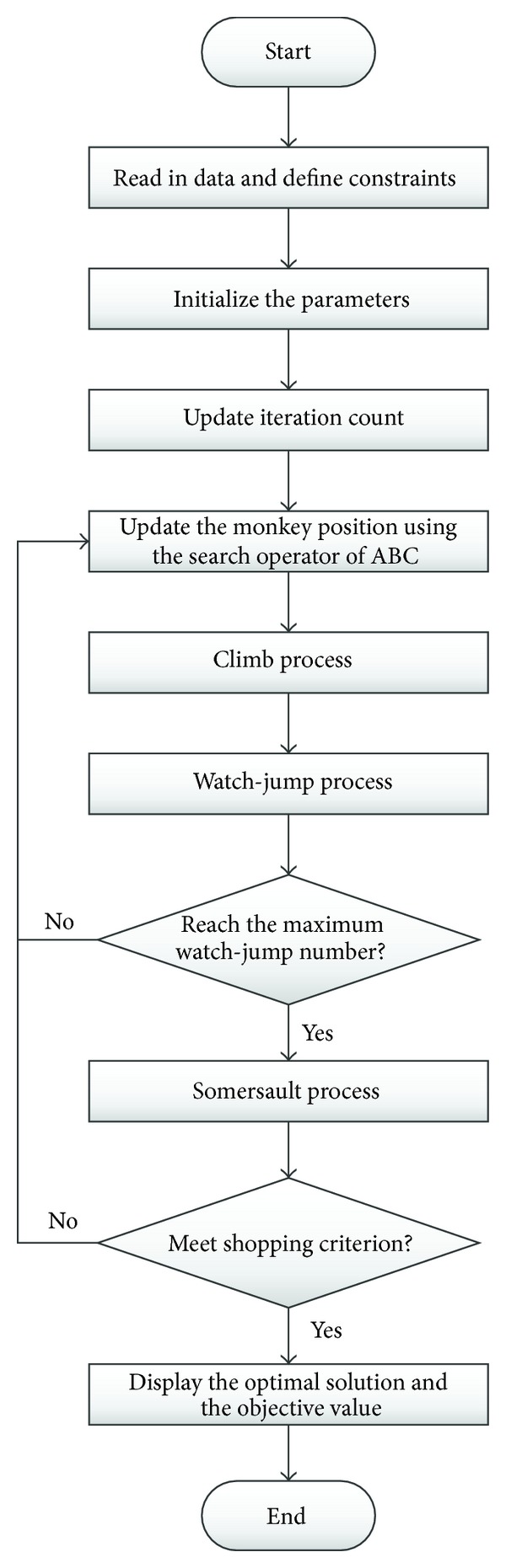
The flow chart of ABC-MA.

To sum up, the whole flowchart of ABC-MA to find the optimal solution of the clustering problem is shown in Figure 3.

## 4. Simulation Experiment

In this section, the experiments were done using a desktop computer with a 3.01 GHz AMD Athlon(tm) II X4640 processor, 3 GB of RAM, running a minimal installation of Windows XP. The application software was Matlab 2012a.

The experimental results comparing the ABC-MA clustering algorithm with six typical stochastic algorithms including the MA [19], PSO [30], CPSO [1, 17], ABC [16, 17], CABC [17], and *k*-means algorithms are provided for two artificial data sets and ten real-life data sets (Iris), Teaching Assistant Evaluation (TAE), wine, seeds, Ripley's glass, Statlog (heart), Haberman's survival, balance scale, Contraceptive Method Choice (CMC), and Wisconsin breast cancer which are selected from the UCI machine learning repository [31].

**Figure 4:**
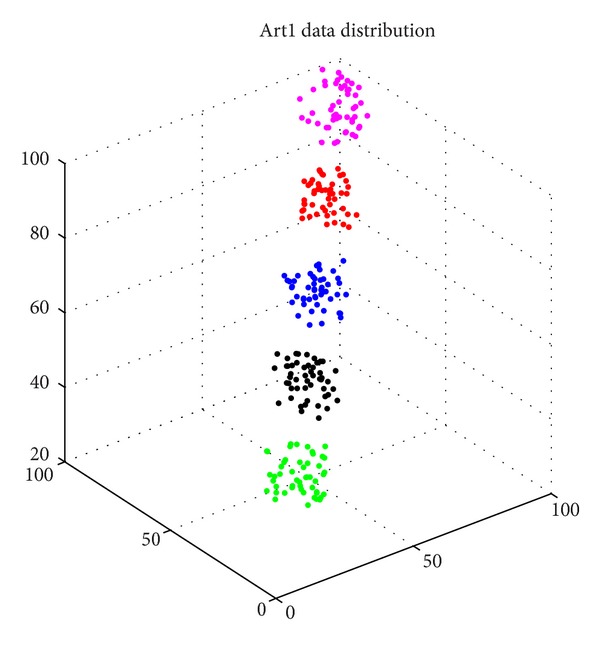
The distribution image of Art1.

Artificial data set one (*N* = 250, *d* = 3, *K* = 5): this is a three-featured problem with five classes, where every feature of the classes was distributed according to Class 1-Uniform (85, 100), Class 2-Uniform (70, 85), Class 3-Uniform (55, 70), Class 4-Uniform (40, 55), and Class 5-Uniform (25, 40) [12, 14]. The data set is illustrated in Figure 4.

Artificial data set two (*N* = 600, *d* = 2, *K* = 4). This is a two-featured problem with four unique classes. A total of 600 patterns were drawn from four independent bivariate normal distributions, where classes were distributed according to

**Figure 5:**
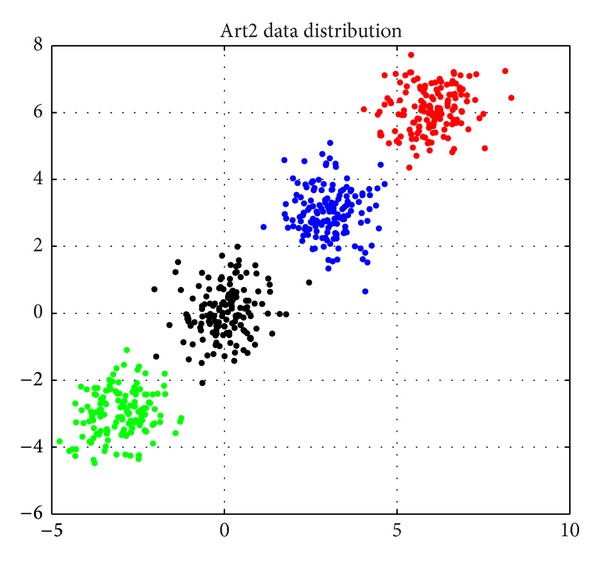
The distribution image of Art2.

(8) N2(μ=(mi0), Σ=[0.50.050.050.5]),

*i* = 1,2, 3,4, *m*_1_ = −3, *m*_2_ = 0, *m*_3_ = 3, *m*_4_ = 6, *μ* and Σ being mean vector and covariance matrix, respectively [12, 14]. The data set is illustrated in Figure 5.

Iris data (*N* = 150, *d* = 4, *K* = 3): this data set with 150 random samples of flowers from the Iris species setosa, versicolor, and virginica were collected by Anderson (1935). From each species there are 50 observations for sepal length, sepal width, petal length, and petal width in cm. This data set was used by Fisher (1936) in his initiation of the linear-discriminant-function technique [14, 17, 31].

Teaching Assistant Evaluation (*N* = 151, *d* = 5, *K* = 3): the data consist of evaluations of teaching performance over three regular semesters and two summer semesters of 151 teaching assistant (TA) assignments at the Statistics Department of the University of Wisconsin-Madison. The scores were divided into 3 roughly equal-sized categories (“low,” “medium,” and “high”) to form the class variable [31].

Wine data (*N* = 178, *d* = 13, *K* = 3): this is the wine data set, which is also taken from MCI laboratory. These data are the results of a chemical analysis of wines grown in the same region in Italy but derived from three different cultivars. The analysis determined the quantities of 13 constituents found in each of the three types of wines. There are 178 instances with 13 numeric attributes in wine data set. All attributes are continuous. There is no missing attribute value [14, 17, 31].

Seeds data (*N* = 210, *d* = 7, *K* = 3): this data set consists of 210 patterns belonging to three different varieties of wheat: Kama, Rosa, and Canadian. From each species there are 70 observations for area *A*, perimeter *P*, compactness *C* (*C* = 4∗*pi*∗*A*/*P*^2), length of kernel, width of kernel, asymmetry coefficient, and length of kernel groove [31].

Ripley's glass (*N* = 214, *d* = 9, *K* = 6): for which data were sampled from six different types of glass: building windows float processed (70 objects), building windows nonfloat processed (76 objects), vehicle windows float processed (17 objects), containers (13 objects), table ware (9 objects), and headlamps (29 objects) each with nine features, which are refractive index, sodium, magnesium, aluminum, silicon, potassium, calcium, barium, and iron [14, 17, 31].

Statlog (heart) data (*N* = 270, *d* = 13, *K* = 2): this data set is a heart disease database similar to a database already present in the repository (heart disease databases) but in a slightly different form [31].

Haberman's survival (*N* = 306, *d* = 3, *K* = 2): the dataset contains cases from a study that was conducted between 1958 and 1970 at the University of Chicago's Billings Hospital on the survival of patients who had undergone surgery for breast cancer. It records two survival status patients with the age of patient at time of operation, patient's year of operation, and number of positive axillary nodes detected [31].

Balance scale data (*N* = 625, *d* = 4, *K* = 3): this data set was generated to model psychological experimental results. Each example is classified as having the balance scale tip to the right, tip to the left, or balanced. The attributes are the left weight, the left distance, the right weight, and the right distance. The correct way to find the class is the greater of (left-distance ∗ left-weight) and (right-distance ∗ right-weight). If they are equal, it is balanced [31].

Wisconsin breast cancer (*N* = 683, *d* = 9, *K* = 2): which consists of 683 objects characterized by nine features: clump thickness, cell size uniformity, cell shape uniformity, marginal adhesion, single epithelial cell size, bare nuclei, bland chromatin, normal nucleoli, and mitoses. There are two categories in the data: malignant (444 objects) and benign (239 objects) [14, 17, 31].

Contraceptive Method Choice (*N* = 1473, *d* = 10, *K* = 3): this data set is a subset of the 1987 National Indonesia Contraceptive Prevalence Survey. The samples are married women who were either not pregnant or do not know if they were at the time of interview. The problem is to predict the current Contraceptive Method Choice (no use, long- term methods, or short-term methods) of a woman based on her demographic and socioeconomic characteristics [14, 17, 31].

Here we set the parameters of ABC-MA and MA as follows: the climb number of ABC-MA *Nc* = 10 and the climb number of MA is set 200, climb step *a* = 0.01, watch-jump number *Nw* = 2, the eyesight *b* = 0.5, somersault interval [*c*, *d*] = [0,2], and the population size *M* = 5. For the PSO, inertia weight *w* = 0.729, acceleration coefficients *c*1 = 2, *c*2 = 2, and population size *M* = 100. The population size of the CPSO is set 20. The population size of the ABC and CABC is set at 50 and 10, respectively. In order to compare with other algorithms, the maximum generations of all algorithms are set at 100.

### 4.1. Algorithm Comparison

For every data set, each algorithm is applied 20 times individually with random initial solution. For the art1 and art2 data set, once the randomly generated parameters are determined, the same parameters are used to test the performance of three algorithms. The best value, the worst value, the mean value, and standard deviation are recorded in Tables 1, 2, 3, 4, 5, 6, 7, 8, 9, 10, 11, and 12. The results are kept four digits after the decimal point.

The simulation results given in Tables 1–12 show that ABC-MA is very precise. As seen from results, the ABC-MA algorithm provides the optimum value and small standard deviation in compare to those obtained by the other methods. For Iris data set, the optimum value, the worst value, the average value, and the standard deviation of ABC-MA are 96.6555, 96.6563, 96.6558, and 3.2699*e* − 04, respectively. CABC also seeks the optimum solution 96.6555, but the standard deviation is bigger than ABC-MA. While the best solutions of MA, ABC, CPSO, PSO, and *k*-means are 96.6614, 96.6566, 96.6580, 96.6556, and 97.1901, respectively.  Table 4 shows the results of algorithms on the TAE dataset. The optimum value is 1490.9258 which are obtained only by ABC-MA. Noticeably other algorithms fail to attain this value even once within 20 runs. The mean value of ABC-MA is 1490.9456 which are smaller than that of MA, CABC, ABC, CPSO, PSO, and *k*-means.  Table 5 provides the results of algorithms on the wine dataset. As seen from the results, the ABC-MA algorithms are far superior to those obtained by the others. For the seeds data set, the best value, the worst value, the worst value, and the standard deviation of ABC-MA are 311.7978, 311.7981, 311.7979, and 1.0051*e* − 04. That means ABC-MA converges to the global optimum value 311.79 in all of runs. The standard deviations for them are 5.6510*e* − 03, 2.1581*e* − 02, 1.0135*e* − 01, 2.8999, and 2.6879*e* − 01, respectively. From the standard deviation, we can see that the ABC-MA algorithm is better than the other methods. For Ripley's glass data set, the optimum value of ABC-MA is 210.0222 which are much better than that of other algorithms. The standard deviations of ABC-MA, MA, and ABC are 8.6924*e* − 01, 7.4809*e* − 01, and 6.9175*e* − 01. On Statlog (heart) dataset results given in Table 8, the best value, the worst value, the worst value, and the standard deviation of ABC-MA are 10622.9824, 10622.9826, 10622.9824, and 3.0810*e* − 05, respectively. It means that the ABC-MA algorithm is able to converge to the global optimum 10622.982 in all of runs, while *k*-means, PSO, and CPSO may be trapped at local optimum solutions. For the Haberman's survival data set, the optimum value 2566.9888 can be obtained by ABC-MA and ABC. But the standard deviation of ABC is 1.2646*e* − 04 which is a little smaller than that of ABC-MA. The standard deviation of PSO is a little smaller than that of CPSO.  Table 10 shows the results of algorithms on the balance scale dataset. As seen from the results, the best value, the worst value, and the mean value of ABC-MA algorithm are much better than those obtained by the others. For Wisconsin breast cancer data set, the best value and the worst value are 2964.3870 and 2964.9883. They are just very close, so the standard deviation is very small. The globe optimal value also can be obtained by the CABC algorithm. But the standard deviation 5.8314*e* − 02 is poorer than that of ABC-MA and MA. On Contraceptive Method Choice data set, the optimum value, the worst value, the average value, and the standard deviation of ABC-MA are 5693.7240, 5693.7418, 5693.7264, and 5.3604*e* − 03, respectively. The best globe solution also can be obtained by the CABC algorithm. The best value and the worst value of PSO are 5766.6412 and 6059.5781. That means PSO may fall into local optimum solutions.

From Table 1 to Table 12, we can conclude that the results obtained by ABC-MA are clearly better than the other algorithms for most of data sets; CABC is a little better than ABC and CPSO is a little better than PSO; the *k*-means is the worst for most of data sets.

Figures 6, 7, 8, 9, 10, 11, 12, 13, 14, 15, 16, and 17 show the convergence curves of different data sets for various algorithms. As seen from the figures, the convergence rate of MA is the fastest. Figures 18, 19, 20, and 21 show the original data distribution of Iris and Haberman's survival data sets and the clustering result by ABC-MA algorithm.

### 4.2. Algorithm Evaluation

In the original MA, the climb step plays a crucial role in the precision of the approximation of the local solution in the climb process. For example, for wine data set, when the climb step is 0.01, the optimum value, the worst value, the average value, and the standard deviation of MA are 16302.7254, 16467.6147, 16366.5331, and 52.4132, respectively. The reason is that the climb step is too small so that sometimes the monkeys cannot arrive at their mountaintops at all in the climb process before the maximal climber number is reached. Here, we replace 0.01 with 0.1 and keep the climb number unchanged. The revised parameters, the optimum value, the worst value, the average value, and the standard deviation are 16293.9147, 16296.2676, 16295.2160, and 5.2270*e* − 01, respectively. The results are better. For the ABC-MA algorithm, the result is not affected by climb step.

In the original MA, the time consumed mainly lies in using the climb process to search local optimal solutions. When we set the climb number 200, it needs computing function values 400 times for every monkey in the climb process. Each iteration needs to calculate about 2000 times function values. For ABC-MA, the computing time is determined by the number of the clusters and the dimensions of the object. For example, for the Iris data set, the number of the clusters is 3 and the dimensions of the object is 4; each iteration needs to calculate the objective values about 160 times which is far less than that of MA. For PSO and ABC, the number of function evaluations is 100 at every iteration, but the results are poor. Because of introducing the cooperative strategy, CPSO [32] and CABC [17] increased a lot of computation time compared with PSO and ABC with the same population size. For example, for Iris data set, when the population size is 100, the numbers of the function evaluations of CABC, CPSO, ABC, and PSO are about 1400, 1300, 200, and 100, respectively. However, CABC and CPSO are difficult to convergence and the result of CPSO is not good.

In order to compare the performance of the three kinds of improved algorithms, the ABC-MA, CABC, and CPSO algorithms are run 20 times individually with 10000 function evaluations. The results are recorded in Table 13. As seen from the results, the results of the ABC-MA algorithm are better than CABC and CPSO. The better solution and the smaller standard deviation can be obtained most of data sets.

**Figure 22:**
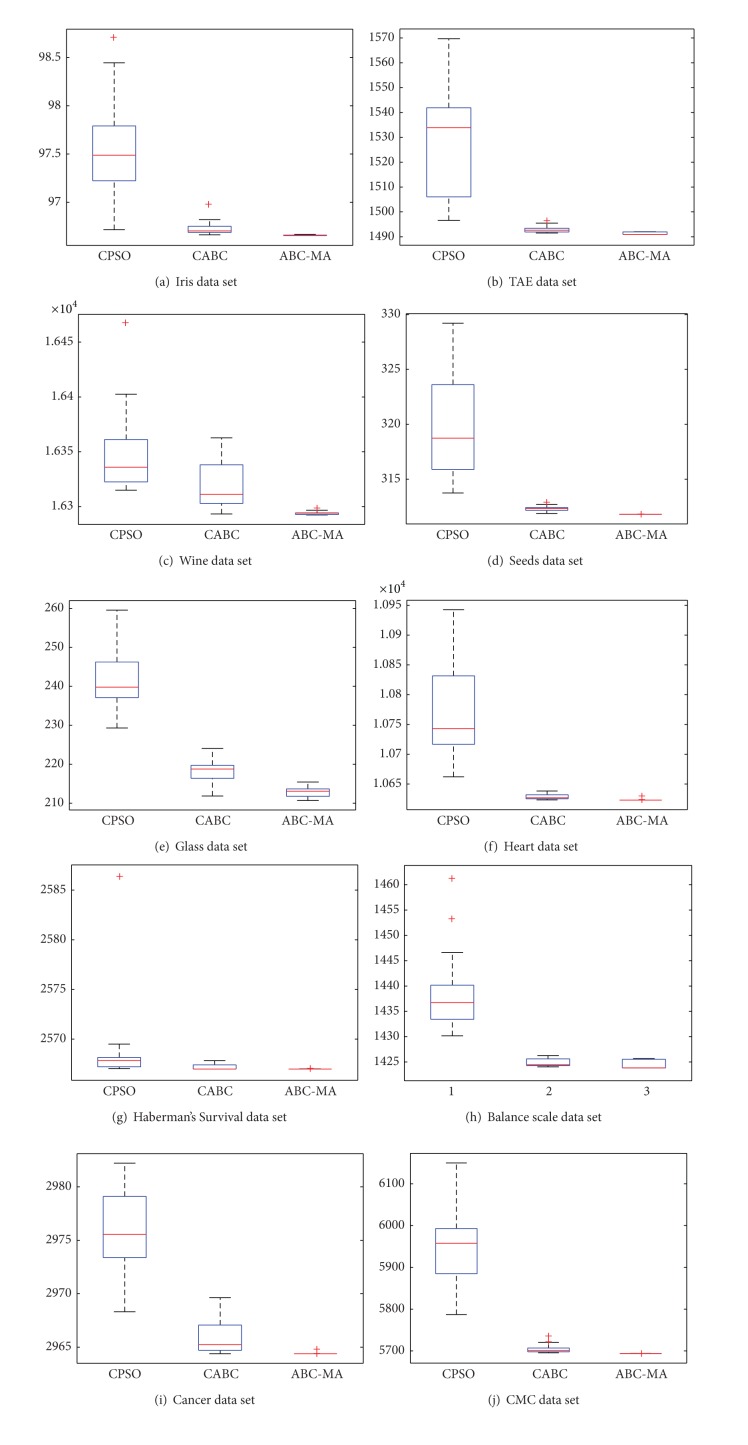
The boxplots of distribution of the objective values obtained by CPSO, CABC, and ABC-MA over 20 independent executions.

The results of CPSO and CABC have apparent difference between the 100 iterations and 10000 function evaluations. However, the difference of ABC-MA is small between the two. We can conclude the ABC-MA has faster convergence speed than CABC and CPSO. The simulation results in the tables demonstrate that the proposed hybrid evolutionary algorithm converges to global optimum with a smaller standard deviation and better globe value and leads naturally to the conclusion that the ABC-MA algorithm is a viable and robust technique for data clustering.  Figure 22 shows The boxplots of distribution of the objective values obtained by CPSO, CABC, and ABC-MA over 20 independent executions. We can see that ABC-MA can obtain smaller upper bound, smaller average, and lower bound of objective values.

## 5. Conclusions

Monkey algorithm is a new swarm intelligence algorithm; its outstanding advantage is that it can effectively avoid falling into local optimal solutions through the somersault process. In the original MA, the precision of the problem is decided by climb step and climb number of the climb process. Because climbing number is large, a lot of running time is consumed in the climb process. In this paper, an improved MA is proposed, artificial colony algorithm search operator is introduced on the basis of the original MA; the local optimal solution can be found by the climb process combined with the artificial colony algorithm search operator, so the climb number is reduced and the running time is far less than the original MA. In view of the clustering problem, we choose the center of objects belonging to the cluster as the pivot to replace the center of all monkeys by the *k*-means algorithm in the somersault process. In this paper, 10 real instances are tested to compare with other algorithms by 100 iterations and 10000 function evaluations. The numerical experiment results show the improved MA has better results than the *k*-means method, PSO, ABC, CPSO, CABC, and MA; especially the testing results of 10000 function evaluations are better, and running time is far lower than the original algorithm. So the improved MA has a good performance than that of the basic monkey algorithm for clustering analysis.

## Figures and Tables

**Figure 6:**
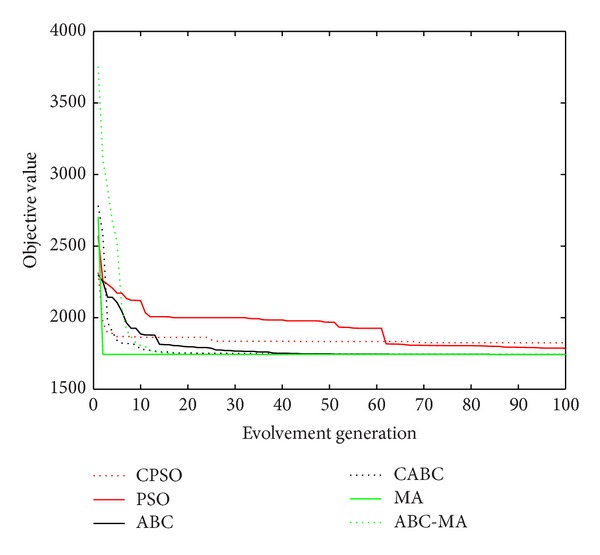
The convergence curve of the Art1 data.

**Figure 7:**
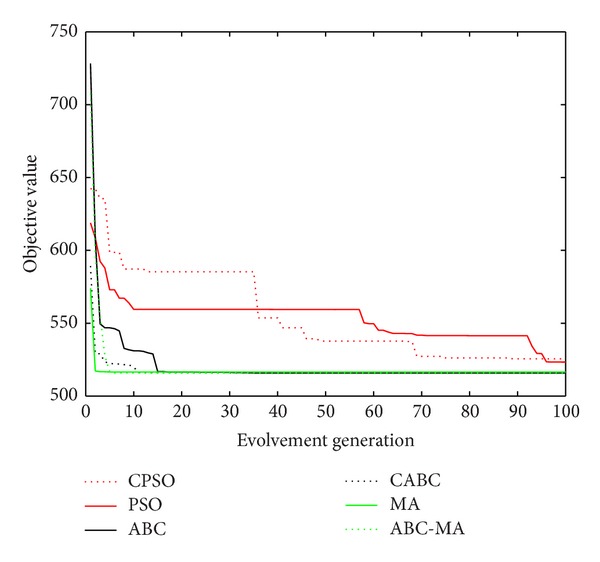
The convergence curve of the Art2 data.

**Figure 8:**
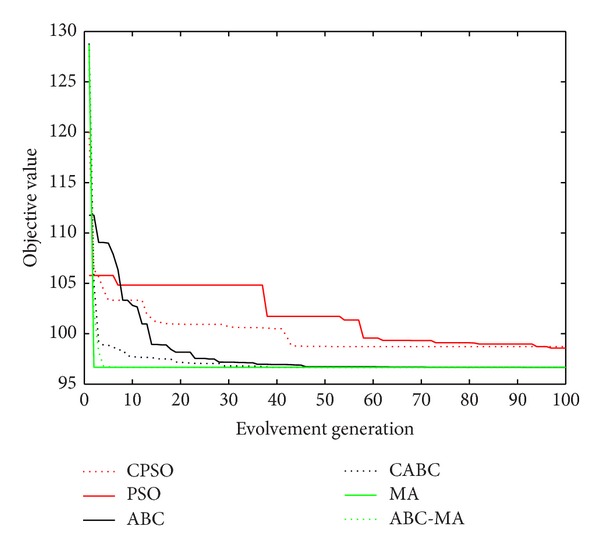
The convergence curve of the Iris data.

**Figure 9:**
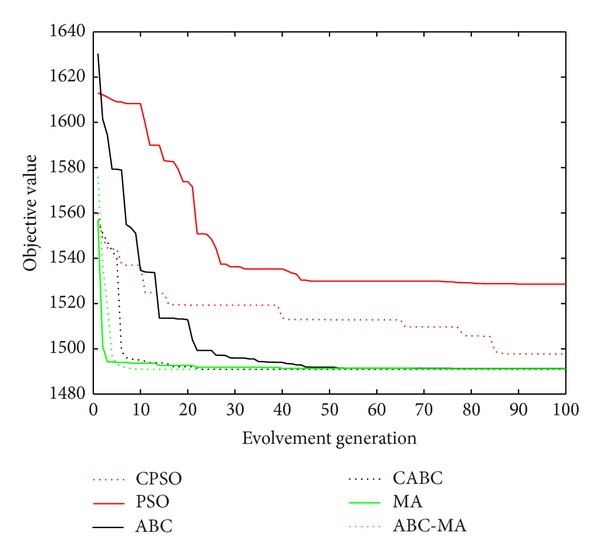
The convergence curve of the TAE data.

**Figure 10:**
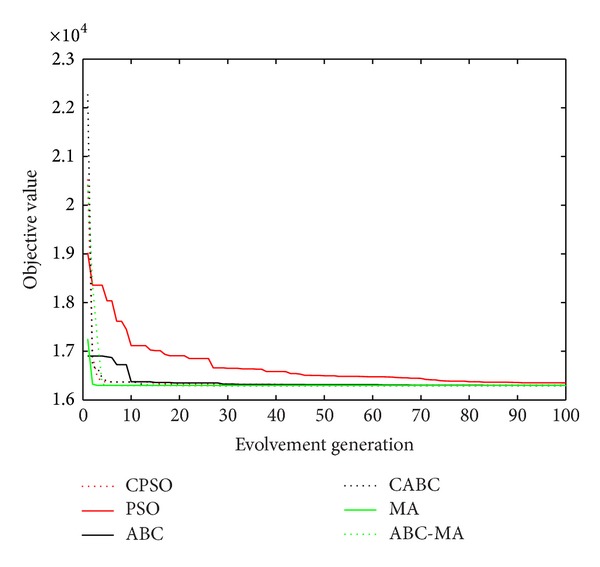
The convergence curve of the wine data.

**Figure 11:**
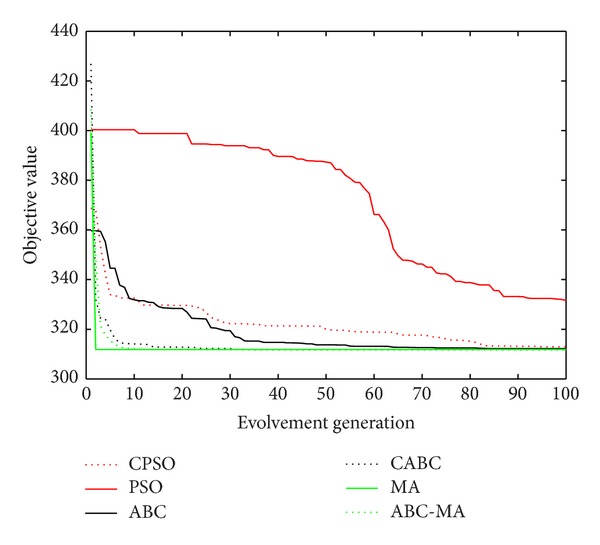
The convergence curve of the CMC data.

**Figure 12:**
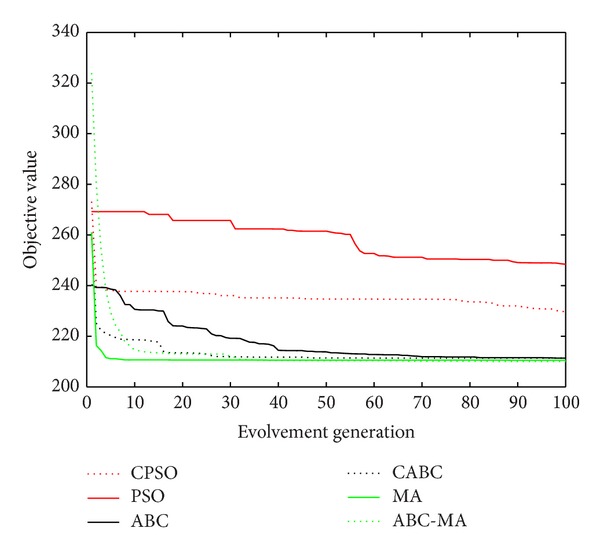
The convergence curve of the seeds data.

**Figure 13:**
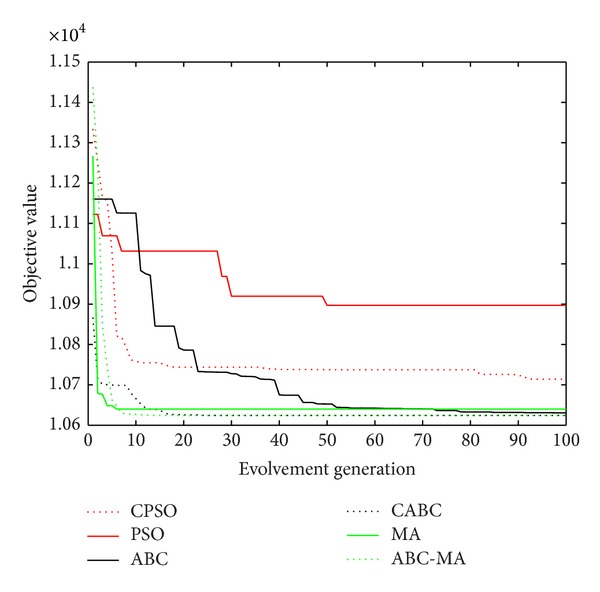
The convergence curve of the glass data.

**Figure 14:**
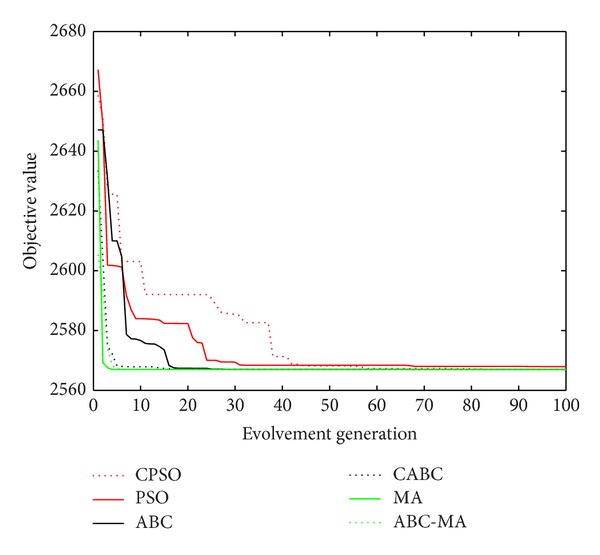
The convergence curve of the heart data.

**Figure 15:**
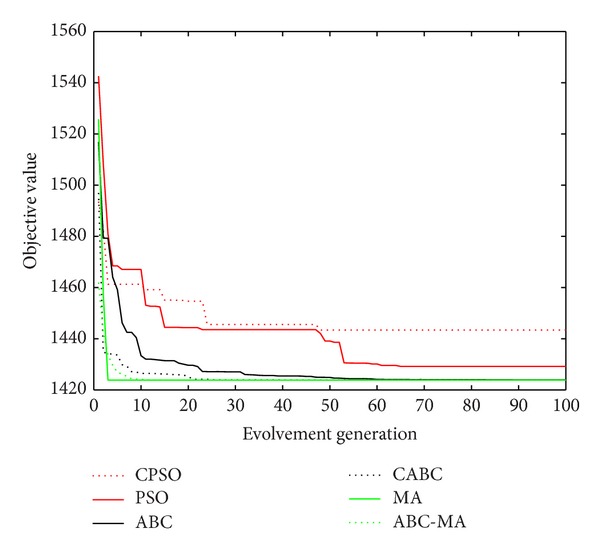
The convergence curve of the survival data.

**Figure 16:**
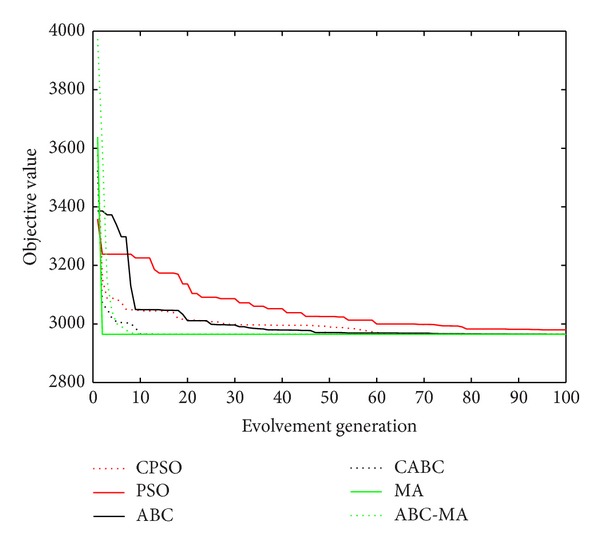
The convergence curve of the scale data.

**Figure 17:**
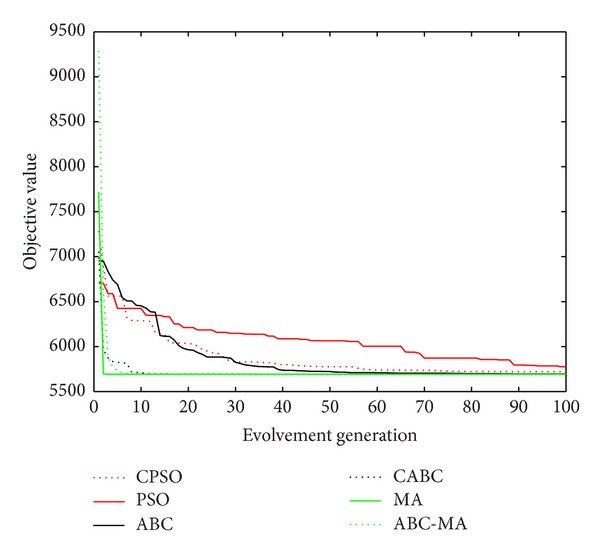
The convergence curve of the cancer data.

**Figure 18:**
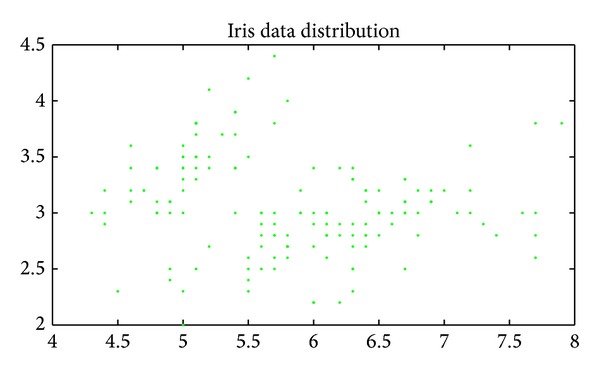
The Iris data distribution.

**Figure 19:**
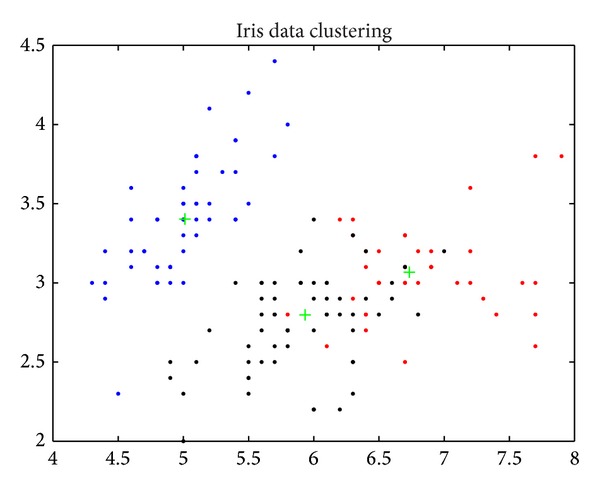
The Iris data clustering result.

**Figure 20:**
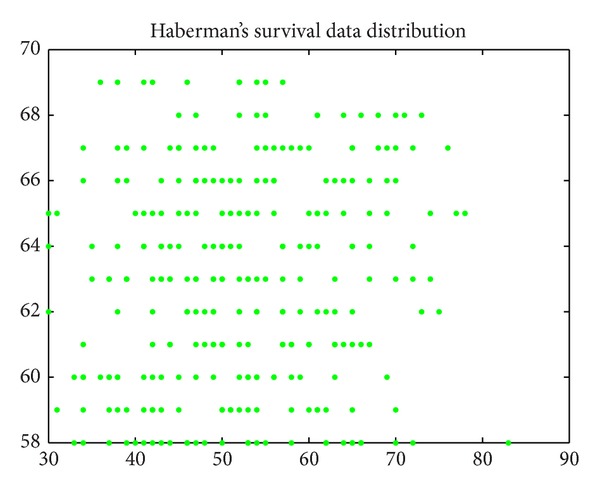
The survival data distribution.

**Figure 21:**
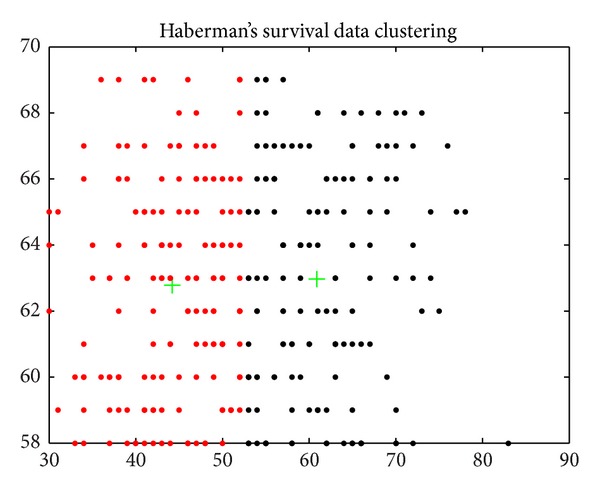
The survival data clustering result.

**Algorithm 1:**
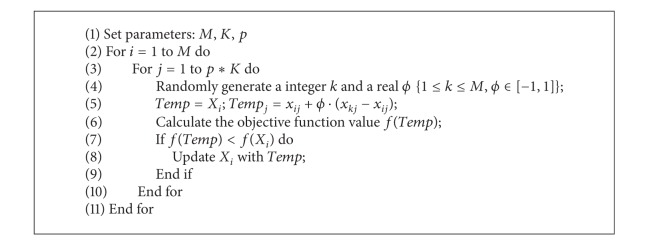


**Table 1:** Results obtained by the algorithms for 20 different runs on Art1 data.

Algorithm	Best	Worst	Mean	Standard
ABC-MA	1743.9470	1743.9486	1743.9478	4.9797*e* − 04
MA	1743.9482	1744.0183	1743.9587	1.8684*e* − 02
CABC	1743.9861	1744.1944	1744.1231	7.1472*e* − 02
ABC	1743.9483	1745.3097	1744.2735	3.2737*e* − 01
CPSO	1746.6026	1996.9473	1866.8777	85.2485
PSO	1829.0508	2259.0049	2015.4896	125.1365
*k*-means	1747.3859	2507.9091	1991.93511	342.2974

**Table 2:** Results obtained by the algorithms for 20 different runs on Art2 data.

Algorithm	Best	Worst	Mean	Standard
ABC-MA	515.7616	515.7628	515.7618	3.5234*e* − 04
MA	515.7635	515.7691	515.7670	1.4338*e* − 03
CABC	515.7616	515.7764	515.7643	3.8176*e* − 01
ABC	515.7616	515.7702	515.7636	2.4925*e* − 01
CPSO	516.6214	529.5846	520.3385	3.9261
PSO	515.9581	571.7869	532.6543	18.6271
*k*-means	525.5957	907.1413	694.4421	191.4831

**Table 3:** Results obtained by the algorithms for 20 different runs on Iris data.

Algorithm	Best	Worst	Mean	Standard
ABC-MA	96.6555	96.6563	96.6558	3.2699*e* − 04
MA	96.6614	96.6685	96.6651	2.0573*e* − 03
CABC	96.6555	96.6599	96.6561	1.1685*e* − 03
ABC	96.6566	96.7547	96.6659	2.1388*e* − 02
CPSO	96.6580	97.5211	96.9721	2.9666*e* − 01
PSO	96.6556	105.1528	99.7345	2.2431
*k*-means	97.1901	121.3554	100.8866	8.7805

**Table 4:** Results obtained by the algorithms for 20 different runs on TAE data.

Algorithm	Best	Worst	Mean	Standard
ABC-MA	1490.9258	1491.0790	1490.9456	3.7515*e* − 02
MA	1491.0358	1491.9663	1491.4607	2.8608*e* − 01
CABC	1490.9276	1492.6488	1491.3099	5.1724*e* − 01
ABC	1490.9808	1491.5794	1491.2134	2.0420*e* − 01
CPSO	1493.3281	1556.9044	1520.8073	21.4859
PSO	1498.6798	1585.0317	1526.7752	25.3170
*k*-means	1504.9535	1603.4106	1529.6406	29.7491

**Table 5:** Results obtained by the algorithms for 20 different runs on wine data.

Algorithm	Best	Worst	Mean	Standard
ABC-MA	16292.1846	16292.6691	16292.2583	1.7657*e* − 01
MA	16302.7254	16467.6147	16366.5331	52.4132
CABC	16292.1849	16294.5850	16292.7695	6.8036*e* − 01
ABC	16293.1685	16310.0568	16298.7961	4.2321
CPSO	16306.2966	16378.3972	16324.8760	18.7122
PSO	16296.4829	16590.2685	16435.4557	71.3194
*k*-means	16325.1202	18436.9520	1745.9957	1003.6327

**Table 6:** Results obtained by the algorithms for 20 different runs on seeds data.

Algorithm	Best	Worst	Mean	Standard
ABC-MA	311.7978	311.7981	311.7979	1.0051*e* − 04
MA	311.8099	311.8378	311.8199	5.3510*e* − 03
CABC	311.7978	311.8947	311.8040	2.1581*e* − 02
ABC	311.8520	312.2110	312.0027	1.0135*e* − 01
CPSO	314.3565	326.2359	318.4564	2.8999
PSO	320.9687	343.4317	332.0422	6.0131
*k*-means	313.1428	313.7343	313.4977	2.6879*e* − 01

**Table 7:** Results obtained by the algorithms for 20 different runs on Ripley's glass data.

Algorithm	Best	Worst	Mean	Standard
ABC-MA	210.0222	212.9732	210.7160	8.6924*e* − 01
MA	210.4653	212.8722	211.6582	7.4809*e* − 01
CABC	210.1789	213.6339	212.4594	1.0456
ABC	210.5709	213.8141	212.3449	6.9175*e* − 01
CPSO	228.4131	251.9513	238.4607	6.3469
PSO	234.5158	254.8014	244.8992	6.1038
*k*-means	215.3043	252.9382	225.4963	12.2847

**Table 8:** Results obtained by the algorithms for 20 different runs on Statlog (Heart) data.

Algorithm	Best	Worst	Mean	Standard
ABC-MA	10622.9824	10622.9826	10622.9824	3.0810*e* − 05
MA	10623.9587	10623.9595	10623.9587	1.7418*e* − 04
CABC	10622.9824	10623.6762	10623.0458	1.5981*e* − 01
ABC	10623.4498	10631.6522	10625.7100	1.9917
CPSO	10649.3132	10747.7609	10688.1370	30.1221
PSO	10671.7870	10935.5974	10787.0485	63.3449
*k*-means	10682.0809	10700.8385	10691.7056	8.2080

**Table 9:** Results obtained by the algorithms for 20 different runs on Haberman's survival data.

Algorithm	Best	Worst	Mean	Standard
ABC-MA	2566.9888	2566.9903	2566.9890	3.4388*e* − 04
MA	2566.9893	2566.9901	2566.9897	2.2211*e* − 04
CABC	2567.0055	2567.9275	2567.3581	3.5868*e* − 01
ABC	2566.9888	2566.9894	2566.9890	1.2646*e* − 04
CPSO	2566.9953	2569.7188	2567.8713	8.5878*e* − 01
PSO	2567.0100	2568.4420	2567.3347	3.9103*e* − 01
*k*-means	2625.1076	3193.5941	2655.1274	126.7500

**Table 10:** Results obtained by the algorithms for 20 different runs on balance scale data.

Algorithm	Best	Worst	Mean	Standard
ABC-MA	1423.8205	1424.1142	1423.8507	9.0004*e* − 02
MA	1423.8243	1423.8306	1423.8267	1.7049*e* − 03
CABC	1423.8206	1424.2445	1423.9109	1.4053*e* − 01
ABC	1423.8308	1424.1153	1423.9238	7.5022*e* − 02
CPSO	1425.4801	1437.6195	1431.8260	2.9772
PSO	1430.4749	1447.6403	1437.1546	4.3708
*k*-means	1423.8514	1434.0441	1426.7539	3.1208

**Table 11:** Results obtained by the algorithms for 20 different runs on cancer data.

Algorithm	Best	Worst	Mean	Standard
ABC-MA	2964.3870	2964.3883	2964.3871	3.1550*e* − 04
MA	2964.4246	2964.4870	2964.4408	1.7096*e* − 02
CABC	2964.3870	2964.5529	2964.4179	5.8314*e* − 02
ABC	2964.5864	2967.2566	2965.1858	6.4821*e* − 01
CPSO	2964.4533	2973.6011	2966.3690	2.4351
PSO	2970.4416	3021.1441	2981.1603	11.1527
*k*-means	2976.9441	2988.4278	2983.3164	4.8661

**Table 12:** Results obtained by the algorithms for 20 different runs on CMC data.

Algorithm	Best	Worst	Mean	Standard
ABC-MA	5693.7240	5693.7418	5693.7264	5.3604*e* − 03
MA	5693.7297	5693.8736	5693.8414	2.9889*e* − 02
CABC	5693.7240	5694.1452	5693.8912	1.5250*e* − 01
ABC	5694.2996	5699.5063	5696.6457	1.4885
CPSO	5699.2901	5739.9530	5709.5340	8.9130
PSO	5766.6412	6059.5781	5906.2983	82.5753
*k*-means	5703.3444	5705.2747	5704.0770	9.0121*e* − 01

**Table 13:** Results obtained by the algorithms for 20 different runs on all data sets.

Data	Algorithm	Best	Worst	Mean	Standard
Art1	ABC-MA	1743.9480	1744.3474	1743.9808	9.7602*e* − 02
	CABC	1744.3744	1806.3827	1761.7068	18.5067
	CPSO	1809.5543	2286.7642	2047.9576	124.0067

Art2	ABC-MA	515.7625	515.7789	515.7666	4.2299*e* − 03
	CABC	515.7630	516.5988	515.9431	2.3931*e* − 01
	CPSO	516.0698	545.4675	528.0076	9.3073

Iris	ABC-MA	96.6555	96.6669	96.6585	3.4742*e* − 03
	CABC	96.6628	97.0046	96.7316	7.8682*e* − 02
	CPSO	96.7162	98.6912	97.5429	5.1969*e* − 01

TAE	ABC-MA	1490.9267	1492.0138	1491.2534	4.9518*e* − 01
	CABC	1491.4669	1496.5907	1492.9128	1.3067
	CPSO	1496.5634	1569.6627	1527.5663	21.1686

Wine	ABC-MA	1629.23445	16297.4038	16293.9540	1.4439
	CABC	16293.2331	16362.7169	16319.2327	21.3326
	CPSO	16314.9904	16466.6525	16348.7576	38.2761

Seeds	ABC-MA	311.7978	311.8233	311.8022	6.0881*e* − 03
	CABC	311.8818	312.9164	312.3053	2.5000*e* − 01
	CPSO	313.7370	329.2068	319.9072	4.5295

Glass	ABC-MA	210.6761	215.4373	212.9199	1.3425
	CABC	211.8449	224.0604	218.1447	2.8634
	CPSO	229.3021	259.5530	241.4084	7.1331

Heart	ABC-MA	10622.9825	10626.6454	10623.1926	8.1864*e* − 01
	CABC	10623.3633	10638.2251	10628.6231	4.5226
	CPSO	10662.0268	10942.4004	10776.0596	78.6768

Haberman's survival	ABC-MA	2566.9889	2567.0176	2566.9935	8.1381*e* − 03
	CABC	2566.9889	2567.8249	2567.1982	3.7121*e* − 01
	CPSO	2567.0174	2586.5513	2568.7706	4.2520

Balance scale	ABC-MA	1423.8243	1425.6662	1424.3613	8.1978*e* − 01
	CABC	1424.0427	1426.2748	1424.8791	7.6287*e* − 01
	CPSO	1430.1559	1461.8830	1438.6882	7.8186

Cancer	ABC-MA	2964.3870	2964.7352	2964.4065	7.7529*e* − 02
	CABC	2964.3870	2969.6338	2965.9663	1.6876
	CPSO	2968.3197	2982.2263	2975.5734	3.8311

CMC	ABC-MA	5693.7360	5694.4571	5693.9155	2.1077*e* − 01
	CABC	5695.5832	5733.9873	5705.0531	10.7000
	CPSO	5787.0284	6149.9020	5945.6423	95.2041
